# Osler-Weber-Rendu Syndrome as a Rare Cause of Liver Cirrhosis

**DOI:** 10.7759/cureus.69869

**Published:** 2024-09-21

**Authors:** Célia Tuna, Teresa Frazão, Sofia Garcês Soares, Maria João Silva, Inês Pinho, José Presa Ramos

**Affiliations:** 1 Internal Medicina Department, Centro Hospitalar Universitário Cova da Beira, Covilhã, PRT; 2 Internal Medicine Department, Hospital da Senhora da Oliveira, Guimarães, PRT; 3 Internal Medicine Department, Centro Hospitalar Tâmega E Sousa, Paredes, PRT; 4 Radiology Department, Centro Hospitalar Trás-os-Montes e Alto Douro, Vila Real, PRT; 5 Internal Medicine Department, Liver Unit, Centro Hospitalar Trás os Montes e Alto Douro, Vila Real, PRT; 6 Internal Medicine Department, Liver Unit, Centro Hospitalar Trás-os-Montes e Alto Douro, Vila Real, PRT

**Keywords:** arteriovenous malformations, clinical case, hereditary hemorrhagic telangiectasia, liver cirrhosis, ultrasound with doppler study

## Abstract

It is essential to comprehend the clinical manifestations of hereditary hemorrhagic telangiectasia (HHT), also known as Osler-Weber-Rendu syndrome. This autosomal dominant vascular disorder presents with distinct symptoms, including mucocutaneous telangiectasia, epistaxis, gastrointestinal bleeding, and iron deficiency anemia. Furthermore, arteriovenous malformations (AVMs) commonly occur in the pulmonary, hepatic, and cerebral circulations. Hepatic involvement may be uncommon, depending on the subtype of HHT. HHT is a rare cause of liver cirrhosis.

We present the case of a 59-year-old female patient referred to a hepatology consultation for the etiological study of liver disease that progressed to liver cirrhosis. From the study carried out, the presence of hepatic arteriovenous shunt was detected, which raised the suspicion of HHT, with the genetic study confirming the diagnosis.

In this case, we intend to demonstrate the diagnostic difficulty of this entity, which led to a long time between the initial manifestation and the diagnostic conclusion. We would also like to highlight the importance of imaging and genetic studies in determining the etiology of the disease.

## Introduction

Hereditary hemorrhagic telangiectasia (HHT), also known as Osler-Rendu-Weber syndrome, is an autosomal dominant disorder with an overall prevalence of at least 1/5000, and given that the symptoms and signs are mild in some cases, the disease is thought to be underdiagnosed [1,2]. It is characterized by telangiectasias of the skin and mucous membranes (often on the tongue, lips, face, ears, and fingers) and arteriovenous malformations (AVMs, more frequent in the lungs, liver, and brain) [3,4]. These malformations are due to the absence of capillaries, resulting in a direct union between arteries and veins [4]. They can also exist at the spinal, gastrointestinal, and pancreatic levels [4]. The presence of AVMs in other less common locations does not govern the diagnosis of HHT [4].

The etiology of this syndrome is related to genetic mutations. Xu et al. report that in 61% of cases, there are mutations in the ENG gene, 37% in the ACVRL1 gene, and 2% mutations in the MADH4 gene [1]. McDonald et al. speak of 52% mutations in the ACVRL1 gene, 44% in the ENG gene, 1% in the SAMD4 gene, and 3% of cases the mutated gene is unknown [4]​​​​​. However, hundreds of genetic mutations have been described [5].

HHT is subdivided into HHT type 1, HHT type 2 and juvenile polyposis, and hereditary hemorrhagic telangiectasia overlap syndrome [2]. This subdivision depends on the genetic mutation involved [2]. Mutations in the ENG gene result in HHT type 1, and mutations in the ACVRL1 gene lead to HHT type 2 and usually present hepatic manifestations of the disease [2]. Mutations in the SMAD4 gene occur in 1 to 2% of cases, leading to disease overlap with polyposis of the gastrointestinal tract [2].

The symptoms of this disease can vary, and its phenotype is partially related to the genetic mutation [2]. Spontaneous and recurrent epistaxis is the most common clinical manifestation and usually begins in childhood [4].

Telangiectasias (except nasal ones) usually appear after episodes of epistaxis, but in most cases, before the age of 40 years old. These vessels, being superficial and fragile, rupture with very mild trauma [4]. In adulthood, the gastrointestinal mucosa is more frequently involved in the stomach and duodenum [4]. This involvement can lead to chronic hemorrhage and iron deficiency anemia due to blood loss from AVMs, such as esophageal varices, which form as a consequence of portal hypertension or hepatic cirrhosis [4,5]. Acute gastrointestinal bleeding is uncommon [4].

Large arteriovenous malformations usually occur in the lungs, brain, pancreas, and liver [5]. At the hepatic level, the prevalence of AVMs is related to HHT subtype 2 and is 2 to 4 times more frequent in females [3,5]. Patients generally develop 3 types of intrahepatic shunts: hepatic artery-hepatic vein, hepatic artery-portal vein, and portal vein-hepatic vein [3,5]. The arteriovenous shunt is the most common subtype and can lead to chronic ischemia, which contributes to the development of liver cirrhosis [5].

Arterioportal shunts are rare and are associated with portal hypertension [5], whereas portosystemic shunts are related to cholangiopathy [6]. It is important to note that other complications, such as mesenteric ischemia and biliary diseases, may arise as a consequence of these intrahepatic AVMs [6].

Only 8% of patients with hepatic involvement have symptoms [7]. Hepatic focal nodular hyperplasia is 100 times more prevalent in this syndrome than in the general population [5].

Atrial fibrillation has an annual incidence of 1.6% in patients with fluid overload due to arteriovenous shunt [5]. The diagnosis of this syndrome is clinical and based on the Curaçao Criteria (spontaneous and recurrent epistaxis, multiple mucocutaneous telangiectasias at characteristic sites, pulmonary, cerebral, or hepatic AVMs, first degree relative to HHT; each parameter scores 1; presenting ≥ a score of 3 makes the clinical diagnosis, a score of 2 raises the diagnostic suspicion, requiring a genetic study to confirm the disease, and a score of 1 excludes the possibility of HHT [4,6].

## Case presentation

A 59-year-old woman with no relevant personal or family history and no drug or toxic habits visited the emergency service several times for generalized abdominal pain in 2009-2010. The analytical study showed alterations in the hepatocellular and cholestatic pattern, and the imaging study by abdominal ultrasound and abdominal CT showed a heterogeneous liver structure with nodules difficult to characterize. Given the findings, the patient was referred to a hepatology consultation to complete the study. In this context, she started follow-up at the consultation after completing an etiological study. From the study carried out, we highlight the result of the abdominal MRI, which revealed multiple irregular areas of non-nodular configuration in the liver with signal alteration, but no neoplasia. Analytically, the condition showed progressive improvement; however, in 2012, she again presented analytical alterations in the hepatocellular and cholestatic patterns. Table 1 shows the analytical results between 2010 and 2022.

**Table 1:** Analytical results between 2010 and 2022 The patient's analytical evolution is presented from the beginning of the follow-up at the hepatology consultation up to the present.

	Reference ranges	2010	2012	2013	2014	2015	2016	2017	2018	2019	2020	2022
Hemoglobin	12–16 g/dL	8.6	16.1	13.5	14.2	15.2	13.9	14.6	13.9	15.2	14.4	14.7
Leukocytes	4–11 × 10^3^/μL	10.9	8.1	4.5	3.9	5.9	5.4	5.0	3.9	5.1	4.6	5.8
Platelets	150–400 × 10^3^/μL	221	276	210	233	224	198	218	209	228	258	158
AST	<35 U/L	37	35	37	34	26	44	26	23	27	27	27
ALT	<33 U/L	65	49	39	31	30	58	26	19	27	23	21
FA	35–105 U/L	221	134	102	88	85	117	79	53	95	102	128
GGT	7–32 U/L	242	263	171	144	95	105	69	61	110	120	178
Total bilirubin	<1.2 mg/dL	1.5	1.2	0.6	0.7	0.5	0.7	0.7	0.6	0.3	0.6	0.5
Direct bilirubin	<0.3 mg/dL	0.7	0.4	0.2	0.2	0.2	0.3	0.2	0.2	0.1	0.3	0.3
INR	<1.2 seg	1.6	1.0	1.1	0.9	1.0		1.0	1.0	1.05	1.03	1.0

During this period, a liver biopsy (2010) was performed, showing signs of chronic cholestasis and intrahepatic cholangiectasis secondary to sclerosing cholangitis. The diagnosis of primary sclerosing cholangitis (PSC) was considered, and treatment with ursodeoxycholic acid (250 mg dose, 1 cp 3 id) was started in 2012, with a slight improvement in the analytical changes (cytolysis and cholestasis parameters) until 2015.

Considering the findings, an MRI cholangiogram was performed (2012), showing no alterations in the bile ducts. Thus, the diagnosis of PSC was called into question. In 2015, she repeated a liver biopsy, which revealed liver tissue almost entirely replaced by fibrosclerotic connective tissue and aggregates of amorphous material compatible with liver pigment. In 2017, she performed a new MRI cholangiogram, which again excluded PSC. After this result, treatment with ursodeoxycholic acid was suspended. In 2018, she performed a liver lesion biopsy, which showed morphological aspects suggestive of wall and biliary cyst content.

**Figure 1:**
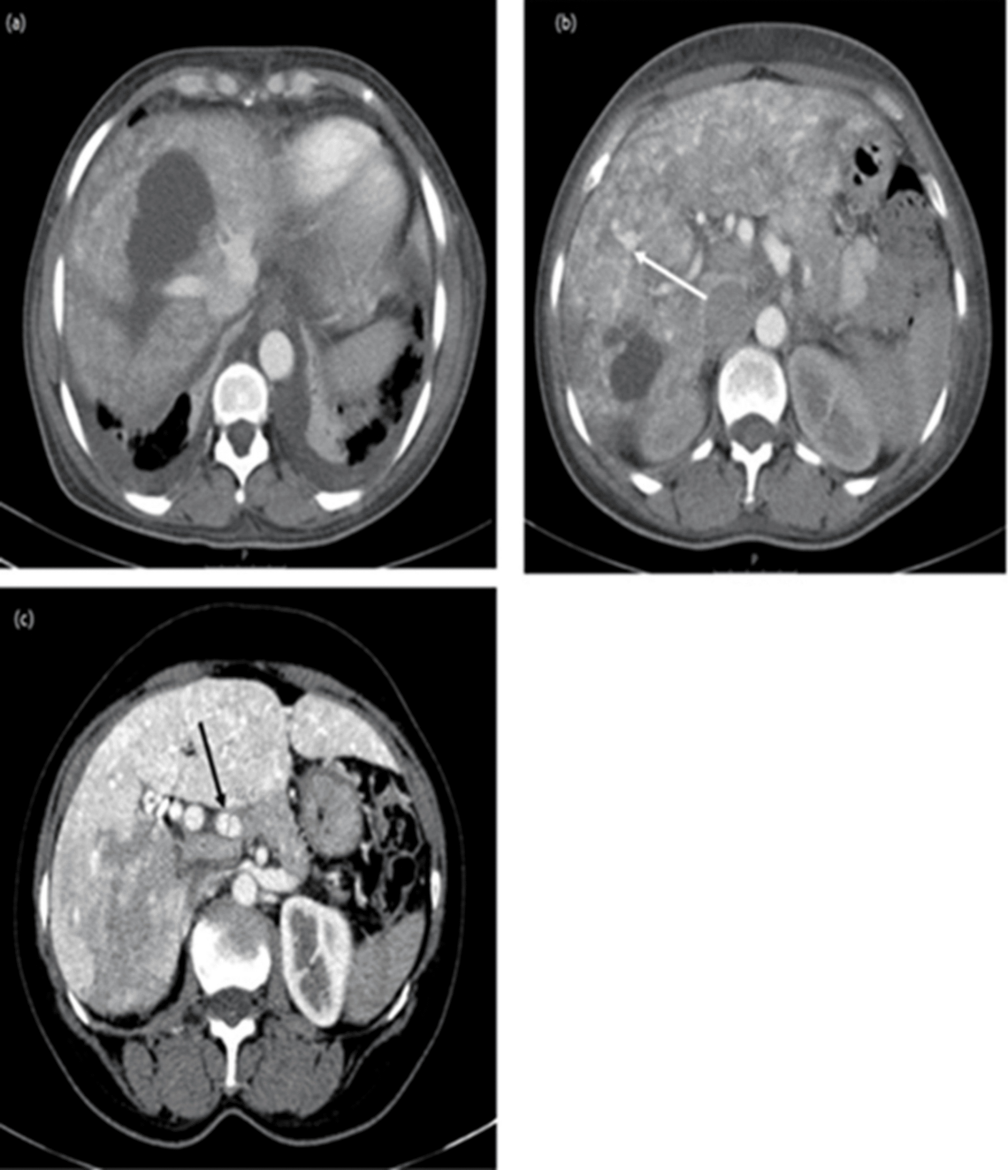
Abdominal CT in arterial phase Liver with signs of chronic liver disease and (b) numerous predominantly peripheraltelangiectasias, as well as arteriovenous shunt or arteriovenous malformations (AVMs) (white arrow). (a) and (b) Hypodense nodular lesions (*), in relation to bile lakes/bilomas. (c) Enlarged hepatic artery (black arrow).

**Figure 2:**
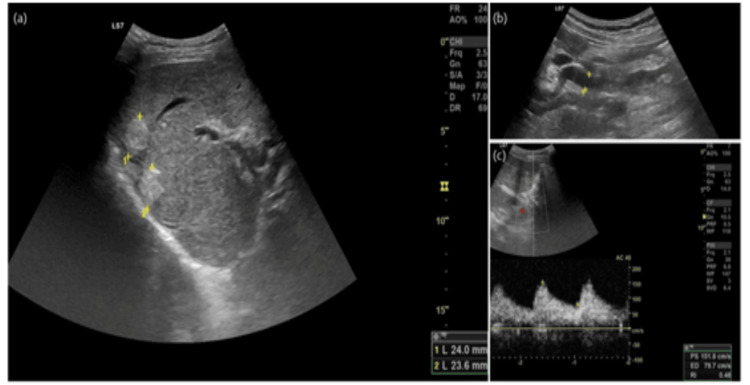
Abdominal ultrasound (a) In liver segment VII, hyperechogenic nodular lesions are identified, without vascularization in the color Doppler study. They translate to bile “lakes”/bilomas (with echogenic biliary debris) resulting from ischemia and biliary necrosis due to hemodynamic changes conditioned by arteriovenous malformations (AVMs); (b) dilated hepatic artery measuring 10 mm in caliber; (c) demonstrating increased velocities (PS: peak systolic velocity > 150 cm/s) and reduced RI: resistance index (0.48) in the Doppler spectral study, hemodynamic alterations resulting from the arteriovenous shunts.

**Figure 3:**
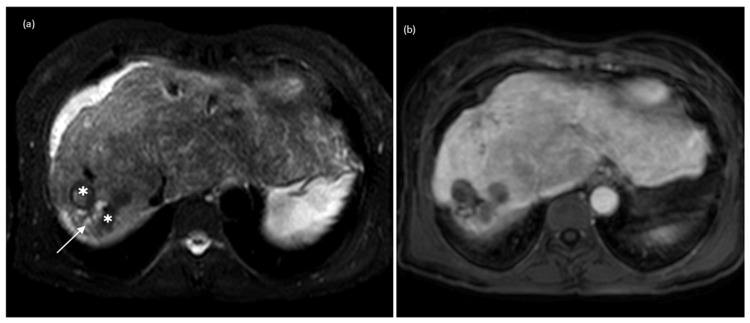
Abdominal magnetic resonance imaging T2-weighted image (a) and dynamic study in portal phase (b). Liver with signs of chronic liver disease. Bilomas are translated by nodular lesions with heterogeneous signal, predominantly hypointense on T2, without flow and/or vascular component in the dynamic study after contrast. Slight ectasia of the intrahepatic biliary tract is also identified upstream of the bilomas (arrows).

During follow-up at the consultation, she performed several imaging tests with contrast-enhanced abdominal CT and abdominal MRI, which showed changes consistent with chronic liver disease. However, only in 2019, a study by a contrast-enhanced abdominal CT showed an increase in the caliber of the hepatic artery and the existence of intrahepatic arteriovenous shunts suggestive of HHT (Figure 1). This change was also observed in the abdominal ultrasound with Doppler (Figure 2) and in the abdominal MRI (Figure 3) performed later.

**Figure 4:**
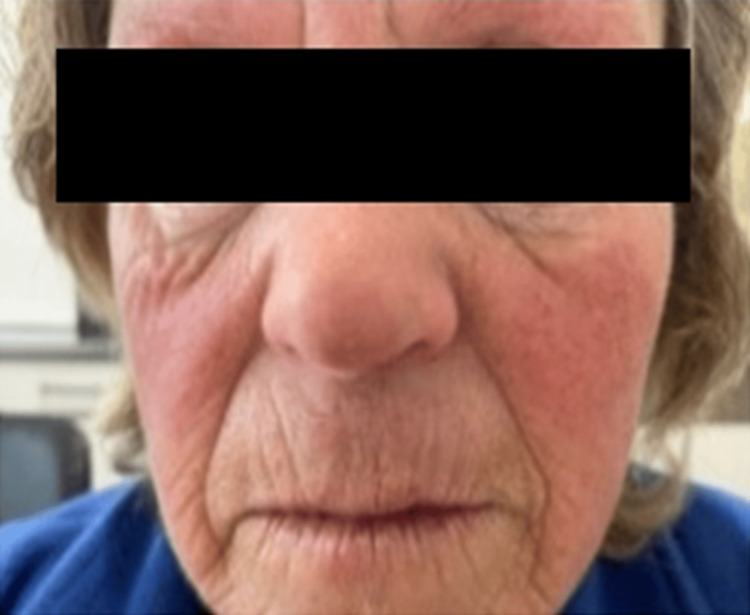
Facial telangiectasias The patient's facial telangiectasias.

On reassessment, she denied epistaxis or a family history of HHT. On physical examination, she presented labial telangiectasias (Figure 4) and scattered cutaneous hemangiomas.

Following the recent examination results, a new assessment was conducted on the patient, and the Curaçao criteria were applied. The patient scored 2 points, attributed to mucocutaneous telangiectasia in the lips and oral mucosa (1 point) and visceral involvement with hepatic arteriovenous malformations (1 point). Since a score of 2 on the Curaçao criteria raises the suspicion of HTT, a genetic study was performed, identifying a heterozygous mutation in the ACVRL1 gene (c.199C>T p. (Arg67Trp)), thus supporting the diagnostic hypothesis. In this context, a complementary study was carried out with chest and skull CT angiography, which ruled out AVMs and transthoracic echocardiograms without alterations in pulmonary artery pressure. Liver disease was staged with hepatic (75 kPa; IQR 0%) and splenic (69.9 kPa; IQR 13%) elastography and upper gastroenteroscopy, which revealed small esophageal varices. According to the recommendations of Baveno VII, carvedilol was started (3.125 mg dose, twice a day) for clinically significant portal hypertension (stage 2).

Currently, she maintains follow-up at the consultation, performing follow-up for patients with liver cirrhosis, with screening for hepatocellular carcinoma, as well as for secondary complications of HHT (such as iron deficiency anemia, high-output heart failure, pulmonary hypertension, and atrial fibrillation). She is currently asymptomatic, without anemia or iron deficiency. Table 2 shows all the causes of liver cirrhosis that were excluded.

**Table 2:** Causes of liver cirrhosis excluded ANA: anti-nuclear antibody; SMA: anti-smooth muscle; AMA: anti-mitochondrial antibodies.

Causes of liver cirrhosis excluded
Chronic viral hepatitis: negative serologies for hepatitis B, C and D.
Alcohol-associated liver disease: no toxic habits.
Hemochromatosis: iron and transferrin saturation normal.
Nonalcohol-associated fatty liver disease: absence of hepatic steatosis by imaging techniques or by liver histology.
Autoimmune hepatitis: ANA, SMA, anti-liver-kidney microsomal antibody (LKM-1) and anti-liver cytosol type 1 (LC-1) negative. Normal gammaglobulinemia levels.
Primary biliary cirrhosis: AMA, ANA (anti-sp100, anti-gp210) negative. Absence of symptoms (including pruritus and fatigue).
Medications: No drug habits.
Wilson disease: 24-hour urinary copper excretion and serum ceruloplasmin normal.
Alpha-1 anti-trypsin deficiency: anti-trypsin alpha-1 normal level.
Infections: Negative serologies for brucellosis, syphilis and human immunodeficiency virus.

## Discussion

In this case, unlike the vast majority of cases, there was no personal history of epistaxis nor a family history of HHT, which made the diagnostic suspicion difficult. Applying the Curaçao criteria (facial and lip telangiectasias and visceral involvement with hepatic arteriovenous malformations), the HHT diagnosis was suspected, and the genetic study confirmed it.

The arteriovenous shunt is often associated with congestive heart failure, hepatomegaly, and pulmonary hypertension [8]. So far, the patient has not experienced any of these complications. Intrahepatic AVMs can lead to the development of cholestasis, which can be anicteric [5,8,9]. This analytical alteration is visible in this clinical case. The MRI cholangiogram did not reveal alterations that would justify the analytical cholestatic pattern, so it can be concluded that these alterations are related to HHT.

In this patient, the performance of contrast-enhanced abdominal CT was essential to raise the diagnostic suspicion. Nevertheless, the ultrasound with Doppler study also showed the alteration in hepatic circulation. Current recommendations suggest that in the presence of HHT or in the suspicion of HHT, AVMs should be investigated with ultrasound with a Doppler study as a first line due to their accuracy, tolerance, safety, and low cost, with multiphase contrast-enhanced CT and MRI being equally useful [9]. Small AVMs should be reassessed every 5 to 10 years, resorting to one of these imaging methods [2]. If the AVM has a feeding artery >2-3 mm in diameter, embolization should be performed [2].

Tsao et al. studied patients with hepatic manifestations of HHT and concluded that chronic ischemia leads to the formation of areas of necrosis and cysts that contain bile [6]. Probably, the nodular areas of difficult characterization evidenced in the imaging exams, as well as the morphology of biliary cysts found on histology, are the result of chronic ischemia caused by the presence of arteriovenous shunts. So far, the patient has not developed anemia or iron deficiency. The guidelines for HHT recommend, from the age of 35, annual measurements of serum levels of hemoglobin and iron, as well as the performance of an endoscopic study (upper endoscopy and colonoscopy) if the anemia value is disproportionate to that of nosebleeds [5]. If the cause of anemia is not found, exploration of the small intestine with capsule endoscopy is recommended [5,9]. In HHT-associated polyposis, there is a risk of intestinal polyps evolving to colon adenocarcinoma, so colonoscopy is recommended from the age of 15 and repeated every three years. Annual screening should be performed if there are polyps or a family history of colon adenocarcinoma. In other types of HHT, the indication for colonoscopy is the same as in the rest of the population. All patients with HHT should be investigated for hemoglobin and iron kinetics regardless of symptoms. In the case of iron deficiency anemia, current recommendations suggest therapy with oral iron unless therapeutic intolerance or anemia is moderate to severe; in this case, intravenous iron therapy or erythrocyte transfusion is necessary, depending on the needs [9].

## Conclusions

We highlight the delay and difficulty in reaching the etiological diagnosis, either because of the rarity of the clinical case or due to the absence of a personal history of epistaxis, the irrelevant family history, and the delay in detecting alterations in imaging studies that would allow the diagnostic suspicion to be raised. We emphasize the essential role of imaging methods (CT and Doppler ultrasound) and the growing importance of genetic studies in clinical practice, as seen in the case reported.
